# Clinical and Pathologic Diagnosis of Renal Lesions

**Published:** 1917-01

**Authors:** 


**Clinical and Pathologic Diagnosis of Renal Lesions.** J. R. Stark, Cin'ti, Jour. of Lab. & Clin. Med., Nov. 600 cases reports with necropsies were studied. In most, the diagnostic terms were obviously used so loosely that only 100 cases were available for analysis and even this number had to be eked out with cases in which it was merely stated that albumin was present, or blood and pus or albumin and casts. The clinical and post mortem diagnoses agrees in 36, but the author seems to regard this as largely due to coincidence and not usually due to distinctive findings in the urinary analysis. They differed in 26. In 37, the clinical diagnosis was not stated but there were indications of some renal abnormality. What was diagnosed as chronic diffuse nephritis proved to be acute in 9 cases, vice versa in 7. 7 cases of tuberculous nephritis were diagnosed as acute twice, chronic twice and as cystitis once, without designation of the tuberculous element. What was diagnosed as tuberculous nephritis proved to be cancer in one case and two cases each called acute parenchymatous nephritis, or simply nephritis, proved to be chronic diffuse nephritis. (Note: It certainly must be admitted that clinical diagnoses often fail to tell the whole truth about lesions present, perhaps largely because opportunities for thorough investigation are so rarely presented by the case while alive. With special reference to urinalyses, it must also be conceded that many of the clinical terms are, so to speak, conventional, even if accurate attempts at naming the disease are made. For instance, the term chronic parenchymatous nephritis signifies, among other things, kidneys that are leaky for certain dyes, that secrete scanty urine, that, in some way are responsible for rather marked dropsy, that furnish broad casts and casts with marked fatty changes—or what are so interpreted. For example, we found such casts in a case, just before death, which was known to have yielded normal urine four days previously, there being no evidence of antecedent disease, and the case obviously being acute. On the other hand, we noted at several examinations in an elderly alcoholic, albumin and various casts. These examinations extended over a period of a couple of years and we certainly believed the case to be one of chronic nephritis. Following an attack of erysipelas, the urine cleared up entirely and was never subsequently found to be abnormal. Some three or four years before the erysipelas the patient had had anuria for a week. How can such a case be explained and properly diagnosed on the basis of any single or few examinations? But, it should be considered, in general, that a post mortem diagnosis is not absolutely the court of last resort. We have seen men without very much experience, slice a kidney, pick at the capsule and record a diagnosis. In contrasting such a diagnosis with one made clinically, odds as to accuracy are even. Ed.)

